# Supplementary material for: ExBoX – a simple Boolean exclusion strategy to drive expression in neurons
Source: J Cell Sci. 2021 Oct 20;134(20):jcs257212. doi: 10.1242/jcs.257212 (PMC8572001; doi:10.1242/jcs.257212)
Supplement: Reviewer comments [file joces-134-257212_review_history.pdf]

ExBoX - a simple Boolean exclusion strategy to drive expression in neurons

Teresa Ubina, Tyler Vahedi-Hunter, Will Agnew-Svoboda, Akshay Gupta, Wenny Wong, Vijayalakshmi Santhakumar and Martin M.Riccomagno

DOI: 10.1242/jcs.257212

Editor: Giampietro Schiavo

Review timeline

Submission to Review Commons:	28 July 2020
Submission to Journal of Cell Science:	12 November 2020
Editorial decision:	20 November 2020
First revision received:	1 September 2021
Accepted:	3 September 2021

Reviewer 1

Evidence, reproducibility and clarity

Manuscript title: ExBoX: a simple Boolean exclusion strategy to drive expression in neurons.

Summary:

In this manuscript, Vahedi-Hunter and co-authors describe and characterize a new set of AAV-compatible vectors allowing conditional expression of cDNA and fluorescent proteins. The reporter system is based on an exclusion system ('AND NOT') by the use of inverted LoxP and FRT sites. Thus, protein expression is turned 'ON' by a recombinase (either CRE or FLP), and 'OFF' by the other recombinase.

The authors describe the construct design and rationale, then demonstrate first in vitro, then in vivo, that the system efficiently works under an exclusion logic. Lastly and as a proof of principle, the authors demonstrate that their vector system can be efficiently used to express activity-modulating proteins (Kir2.1, NaChBac, or TeTN) in the mouse hippocampus.
Before commenting the data and conclusions, it is worth noting that this paper reads easily. The manuscript is well written, and figures are good in quality. The rationale and experimental design are well explained and easy to follow.

Major comments:

The main purpose of this manuscript is to describe and validate a novel expression system called ExBoX. As such, the main conclusion of the paper is that ExBoX efficiently works on an exclusion logic, and this is indeed largely supported by data. Throughout the figures, the authors convincingly show that the Cre-ON;Flp-OFF vector only provides expression when CRE is expressed alone. Conversely, the Cre-OFF;Flp-ON vector provides expression when FLP is expressed without CRE.

The characterization of the system relies on the quantification of GFP and/or Tomato expressing cells in various experimental design. It is worth noting that the authors chose to co-transfect (or co-electroporate) ExBoX vectors with vectors expressing CRE, FlpO, and an additional fluorescent protein under a constitutive promoter. Thus, the quantification relies on the relative GFP (ExBoX) / RFP (constitutive) expression, assuming 100% co-transfection and expression of all 4 plasmids.

© 2021. Published by The Company of Biologists under the terms of the Creative Commons Attribution License (https://creativecommons.org/licenses/by/4.0/).

Staining of CRE and/or Flp by immunofluorescence would convincingly demonstrate co-expression of recombinases in all cells and strengthen the data.

Another important aspect of ExBoX characterization is the temporal resolution of the system. Fate-mapping experiments using an exclusion system are a powerful tool to access temporal windows associated to specific developmental events, for example. This is largely discussed by the authors but one can regret that these experiments are not included in this manuscript. The model presented in Figure 8 could have easily been demonstrated using the FlpON- CreOff-TdTomato ExBoX vector expressed in any CRE-ERT2 mouse or cell line, together with constitutive FlpO expression. Thus, the authors would be able to easily demonstrate and quantify signal extinction after CRE induction by tamoxifen. Actually knowing if Tomato is turned off in a matter of hours, or in a matter of days, would make an important difference for the feasibility of temporally-controlled fate-mapping experiments. Thus, this additional experiment is technically feasible and would increase the impact of the paper.

Finally, experiments in Figures 5-7 are not quantified. It appears that there are a few escapers from the exclusion system in vivo: see for example Figure 5F, figure 6F and Figure 7S-T. The authors should discuss this, and quantify the % of cells remaining ON. Do these escapers cells result from incomplete co-infection with CRE and FlpO AAVs? For example in Figure 5F even a 95% co-infection rate would result in a few Tomato+ neurons. This can be detected by CRE immunostaining to check if the Tomato expressing neurons are CRE negative. Alternatively, this could stem from late recombination by the OFF recombinase, and slow elimination of the fluorescent protein.

Minor comments:

-The authors used a synapsin promoter whith is a neuron-specific, weak and late promoter. If I understand correctly immune-amplification of the signal with anti-GFP and anti-DsRed has been applied to in vivo experiments. The authors could clarify this point and maybe provide one image without amplification so future users of ExBoX can evaluate the feasibility of in vivo experiments without amplification (ie. live imaging, etc...)

-Table 1: the left column could be more clear. There's a confusion between recombinase expression, as indicated in the first line (column header) and the vector name/experimental system, referenced in the first column. Using the same nomenclature as in the manuscript (CreON-FlpOff-EGFP ExBoX, and FlpON-CreOff-TdTomato ExBoX) would be clearer.

-Figure 1 and 2: vector design representation could be improved. I would strongly suggest to use distinct symbols for Lox and Frt sites. Also being consistent in color scheme is important: Figure 1A, black arrowheads are Frt sites, but Figure 2A, black arrowheads are Lox2722 sites. Figure 1A, LoxP and Lox2722 sites are represented as red and blue arrowheads, yet CRE expression/recombination is indicated as a black arrow. Frt sites are represented as black arrowheads, yet FlpE expression/recombination is indicated ad a red arrow. Figure 1A, CRE is black arrow and FlpE is red arrow, yet Figure 2A, CRE is red arrow and FlpE is black arrow.

-Figure lettering seems to be mixed in the first paragraph of 'Validation of ExBoX AAVs in vivo' (figure 5).

Significance

The purpose of ExBoX is to provide a simple and efficient exclusion logic (AND NOT) reporter system. The authors make it clear that this strategy has been developed previously and thus ExBoX is not a novel method but rather a refinement, providing a simpler construct design that might be easier to use. While this reviewer does not have any hands-on experience with AAV vectors use and virus production, the author's argument seem valid and thus my impression would be that ExBoX provides some technical advance and would be useful for the scientific community.

Of note the authors validate ExBoX in neurons, but the same system could easily be adapted to other tissues simply by changing the promoter of the ExBoX construct. Thus, the system might be of interest outside of the neuroscience community.

© 2021. Published by The Company of Biologists under the terms of the Creative Commons Attribution License (https://creativecommons.org/licenses/by/4.0/).

Expertise of the reviewer: the reviewer has expertise in mouse cortical development and neurobiology, as well as with gene-expression and manipulation techniques in the mouse brain.

Reviewer 2

Evidence, reproducibility and clarity

Overall this is not a novel method and design of cell type targeting. So it depends on what level of significance this paper is aimed to be published.

I have not much to comment on. The design is straight-forward, but also very similar to the conditional TRAP design used in previous conditional and reversible gene regulation papers (links provided below). The concept is essentially the same except a reporter gene instead of a TRAP cassette was flanked by the (f)DIO-switches, and it is used in virus instead of mouse genome. https://www.ncbi.nlm.nih.gov/pmc/articles/PMC4635517/(Fig 1b; Identical design if the splicing acceptor before the GFP is removed?)

https://www.nature.com/articles/nmeth.4156 (Supplementary Fig. 11a)
I would also suggest them to cite this paper (https://www.cell.com/cell/pdf/S0092-8674(20)30047-7.pdf) in which the same concept (inversion+deletion) was used to design Cre-on-Flp-off AAV . Instead of DIO switches, the lox66-lox71 pair to achieve irreversible inversion mediated by Cre in that paper. The efficiency would be lower than DIO but the idea is the same.

Significance

N/A

Reviewer 3

Evidence, reproducibility and clarity

Their idea seems really solid and they did a great job validating it. All the date are really clean

Significance

Here the authors have created and validated a tool for greater precision over transgene expression. While a similar tool exists, this system is more ideal for viral packaging which is vital for use in vivo. Combining Cre and FLP, and theoretically others, this system works as Boolean operators to express or not express genes of interest. The authors have thoroughly validated their tool in culture models and viral injections in mouse brains.

I have no major concerns and would be happy to see this tool disseminated

---

Author response to reviewers' comments

Response to Reviewers

My colleagues and I thank you for considering our manuscript, “ExBoX: a simple Boolean exclusion strategy to drive expression in neurons,” for publication. We are pleased that the reviewers found that the manuscript is “*well written* “, the study is “*really solid*”, the main conclusions are “*indeed largely supported by data*”, and the tools developed will be “*useful for the scientific community*”. We are also glad that reviewers were positive about the quality of data and thoroughness of the validation process, and are pleased that they raised no major concerns. I also would like to express our appreciation to the reviewers for their thoughtful comments and

© 2021. Published by The Company of Biologists under the terms of the Creative Commons Attribution License (https://creativecommons.org/licenses/by/4.0/).

suggestions—attention to the outstanding issues will greatly improve this study. Below are our responses to the reviewers' comments, including discussion of points raised and, in many cases, additional data that will be provided in the revised manuscript. The most important addition to the revised manuscript will be a proof-of-concept experiment to validate the use of ExBox to access developmental windows (see point 2 below), an experiment that as Reviewer 1 states "*would increase the impact of the paper*".

Reviewer 1
We thank this Reviewer for their positive comments on our study, including: *"… it is worth noting that this paper reads easily. The manuscript is well written, and figures are good in quality. The rationale and experimental design are well explained and easy to follow.*", and that "*the main conclusion of the paper is that ExBoX efficiently works on an exclusion logic, and this is indeed largely supported by data*." We have addressed this reviewer's comments, suggestions and minor points below.

1) The characterization of the system relies on the quantification of GFP and/or Tomato expressing cells in various experimental design. It is worth noting that the authors chose to co-transfect (or co-electroporate) ExBoX vectors with vectors expressing CRE, FlpO, and an additional fluorescent protein under a constitutive promoter. Thus, the quantification relies on the relative GFP (ExBoX) / RFP (constitutive) expression, assuming 100% co-transfection and expression of all 4 plasmids. Staining of CRE and/or Flp by immunofluorescence would convincingly demonstrate co-expression of recombinases in all cells and strengthen the data.

We agree with the reviewer in that this would be a nice addition to the data. It is worth noting that, for Figures 1 through 4 all the constructs expressing the OFF recombinases are bicistronic and express a fluorescent reporter from the same promoter. This was not very clear in the manuscript, except for the legend for Figure 4. We will now make this clearer in the main text and the other figure legends. This should address part of the concern raised by the reviewer.

In addition, we are currently trying to stain for Cre and Flp in different experimental settings to detect the ON recombinase. Unfortunately, we are limited by the reagents currently available to visualize these recombinases. We have had limited success with Cre antibodies, but expect to be able to detect it at least in cell lines. In terms of FlpO, we haven't had any success, but we are testing an additional antibody. We would like to provide these additional data in a revised manuscript, if it becomes available.

It is nevertheless important to note that if the co-transfection were inefficient, we would be underestimating the number of cells expressing the constructs in the ON condition. When we co-transfect ON recombinase + loading fluorescent reporter with the CreON- FlpOFF or FlpON-CreOFF constructs we detect co-expression in about 90 to 100% of the cells expressing the loading reporter (Figures 1-4). This suggests 2 things:

a) That the activation of the ExBox constructs is extremely high.
b) The co-transfection efficiency is high, otherwise the proportion of cells expressing both reporters would be much lower.

2) Another important aspect of ExBoX characterization is the temporal resolution of the system. Fate-mapping experiments using an exclusion system are a powerful tool to access temporal windows associated to specific developmental events, for example. This is largely discussed by the authors but one can regret that these experiments are not included in this manuscript. The model presented in Figure 8 could have easily been demonstrated using the FlpON-CreOff-TdTomato ExBoX vector expressed in any CRE- ERT2 mouse or cell line, together with constitutive FlpO expression. Thus, the authors would be able to easily demonstrate and quantify signal extinction after CRE induction by tamoxifen. Actually knowing if Tomato is turned off in a matter of hours, or in a matter of days, would make an important difference for the feasibility of temporally-controlled fate-mapping experiments. Thus, this additional experiment is technically feasible and would increase the impact of the paper.

This is a great suggestion. We had already started testing this using a newly developed AAV-Ef1a-FlpOERT2 with an improved linker. Preliminary data suggest an almost complete inactivation of CreON-FlpOFF-EGFP reporter 3 weeks after tamoxifen gavage. This is an important proof-of-

© 2021. Published by The Company of Biologists under the terms of the Creative Commons Attribution License (https://creativecommons.org/licenses/by/4.0/).

principle experiment showing the power of the ExBox tools. We are in the process of trying the same experiment detecting expression a couple of days or 1 week after tamoxifen, and plan to present the results in the revised manuscript. Even with a highly efficient system, it is unlikely that protein expression will be turned off within a few hours, but this will be ultimately dependent on mRNA and protein stability for each gene of interest. Thus, we think our system will be most powerful when studying developmental events that take place over days or weeks.

3) Finally, experiments in Figures 5-7 are not quantified. It appears that there are a few escapers from the exclusion system in vivo: see for example Figure 5F, figure 6F and Figure 7S-T. The authors should discuss this, and quantify the % of cells remaining ON. Do these escapers cells result from incomplete co-infection with CRE and FlpO AAVs? For example in Figure 5F even a 95% co-infection rate would result in a few Tomato+ neurons. This can be detected by CRE immunostaining to check if the Tomato expressing neurons are CRE negative. Alternatively, this could stem from late recombination by the OFF recombinase, and slow elimination of the fluorescent protein.

We agree with the reviewer in that we can provide better quantification for those experiments. In addition to presenting the loss of relative fluorescence as in the supplemental figures, we will now present the % of escapers for each of the ON+OFF conditions. We will try co-staining for the recombinases, but as stated before, we have had a hard time detecting them, especially FlpO. Furthermore, we are conducting some additional proof-of-concept experiments where we co-express an additional fluorescent reporter to better normalize the number of cells expressing the ExBox constructs in the ON vs. the OFF+ON conditions.

Minor comments
We found all these comments very valuable and will address them by editing the text and/or figures.

Significance:
The purpose of ExBoX is to provide a simple and efficient exclusion logic (AND NOT) reporter system. The authors make it clear that this strategy has been developed previously and thus ExBoX is not a novel method but rather a refinement, providing a simpler construct design that might be easier to use. While this reviewer does not have any hands-on experience with AAV vectors use and virus production, the author's argument seem valid and thus my impression would be that ExBoX provides some technical advance and would be useful for the scientific community. Of note the authors validate ExBoX in neurons, but the same system could easily be adapted to other tissues simply by changing the promoter of the ExBoX construct. Thus, the system might be of interest outside of the neuroscience community.

We would like to thank the reviewer for stating that ExBox could provide some "*technical advance and would be useful for the scientific community*", and that "*could easily be adapted to other tissues simply by changing the promoter of the ExBoX construct. Thus, the system might be of interest outside of the neuroscience community.*"

We are in agreement with the reviewer in that similar strategies were designed before, and as stated by them, we make this clear through-out the paper. The goal of our new ExBox strategy was to make the use of these tools universal, by providing a simpler design and a more efficient use of vector space. In addition to redesigning a Boolean exclusion system we generated new viral CreOn-FlpOff constructs for modulation of neuronal activity using Kir2.1, NachBac and TenT. Finally, we generated a new FlpOERT2 AAV that will have broad applicability. We couldn't find a viral vector like that in any public repository. All these constructs are being deposited in Addgene and will be available to the scientific community. Additional considerations about impact and novelty are discussed in response to Reviewer 2.

Reviewer 2
We thank reviewer 2 for their comments. We were glad to see that this reviewer didn't have any technical or methodological concerns. We also thank the reviewer for bringing those studies to our attention. We will now cite them in the main text of the manuscript.

© 2021. Published by The Company of Biologists under the terms of the Creative Commons Attribution License (https://creativecommons.org/licenses/by/4.0/).

The only issue raised by Reviewer 2 was about novelty. We tried to make it clear through-out our manuscript that we are building on previous work (Fenno et al., 2014 among other citations) to design a simpler, more efficient and universal tool to drive tightly controlled expression using Boolean exclusion logic. By using viruses with a very simple modular design we believe we achieved this goal. Anyone with any interest in using these tools can clone their gene of interest in these vectors in a simple step. In addition, promoters can be easily replaced to drive expression in a multiplicity of tissues and model organisms for a very low cost. This also adds to the universality of the tools. Furthermore, as mentioned in the discussion, the modularity of the system would make it easy to adapt to other recombinases (e.g. DRE), or to add additional sites to achieve an AND NOT NOT logic. Finally, we also introduce new viral CreOn-FlpOff constructs for Kir2.1, NachBac and TenT, and a new FlpOERT2 AAV that will have broad applicability. Taken together we believe that the importance of such work in driving science forward resides not only in adding "new arrows to the quiver", but in making them easier to use, easier to build upon, easier to disseminate, and easier to adapt to different animal models and organs.

That said, the design of the ExBox AAVs is actually novel. To our knowledge, there are no published or publicly available AAVs using this strategy. We truly believe that the papers cited by Reviewer 2 don't take anything away from the impact of our work. The two papers describing the generation of TRAP tools, while using a very similar design to our CreOn-FlpOff construct, involve the generation of *knock in* mice, and the overall goal of the approach is exactly the opposite of that of ExBox: control the silencing of endogenous genes. In addition, the design of the FlpOn-CreOff constructs is completely new. We will cite these two papers in the introduction as examples of combinatorial approaches. The third paper cited by Reviewer 2, which was published while this manuscript was in preparation, uses an elegant but different design (a hybrid *lox* system) to achieve something very similar to what we propose. However, as stated by the reviewer, it is expected that ExBox system would be more efficient (in addition to being more modular, more widely applicable and adaptable). This will now be mentioned in the discussion section.

Reviewer 3
We are very pleased with the extremely positive comments this reviewer has made on the impact, significance, and quality of our study. We agree with Reviewer 3 in that ExBox is "*ideal for viral packaging which is vital for use in vivo*."

It is our intention to disseminate these tools for immediate use by the scientific community. We have already started the process of depositing them at Addgene.

Original submission

First decision letter

MS ID#: JOCES/2020/257212

MS TITLE: ExBoX: a simple Boolean exclusion strategy to drive expression in neurons

AUTHORS: Tyler Vahedi-Hunter, Teresa Ubina, Will Agnew-Svoboda, Akshay Gupta, Wenny Wong, Vijayalakshmi Santhakumar, and Martin Miguel Riccomagno
ARTICLE TYPE: Tools and Resources

We have now reached a decision on the above manuscript based on the comments of the reviewers and your response to their criticisms.

Whilst reviewer 3 does not suggest any change to the manuscript, Reviewers 1 and 2 expressed technical and novelty concerns, respectively. Whilst a better description on how the current system differs from published methods will suffice to address the second reviewer, additional experiments as canvassed in the Reviewers' response are deemed necessary. In particular, I encourage you to

© 2021. Published by The Company of Biologists under the terms of the Creative Commons Attribution License (https://creativecommons.org/licenses/by/4.0/).

incorporate in the revised manuscript a proof of CRE staining, a quantification of the data shown in Figure 5 -7 and the kinetic of inactivation of this genetic switch in vivo.

We are aware that you may be experiencing disruption to the normal running of your lab that makes experimental revisions challenging. If it would be helpful, we encourage you to contact us to discuss your revision in greater detail. Please send us a point-by-point response indicating where you are able to address concerns raised (either experimentally or by changes to the text) and where you will not be able to do so within the normal timeframe of a revision. We will then provide further guidance. Please also note that we are happy to extend revision timeframes as necessary.

Please ensure that you clearly highlight all changes made in the revised manuscript. Please avoid using 'Tracked changes' in Word files as these are lost in PDF conversion.

I should be grateful if you would also provide a point-by-point response detailing how you have dealt with the points raised by the reviewers in the 'Response to Reviewers' box. Please attend to all of the reviewers' comments. If you do not agree with any of their criticisms or suggestions please explain clearly why this is so.

First revision

Author response to reviewers' comments

Response to Reviewers

My colleagues and I thank you for considering our manuscript, “ExBoX: a simple Boolean exclusion strategy to drive expression in neurons,” for publication. We are pleased that the reviewers found that the manuscript is “*well written* “, the study is “*really solid*”, the main conclusions are “*indeed largely supported by data*”, and the tools developed will be “*useful for the scientific community*”. We are also glad that reviewers were positive about the quality of data and thoroughness of the validation process, and are pleased that they raised no major concerns. I also would like to express our appreciation to the reviewers for their thoughtful comments and suggestions—attention to these outstanding issues greatly improved the study. Below are our responses to the reviewers’ comments, including discussion of points raised and, in many cases, extensive additional data that is provided in the revised manuscript. The most important additions to the revised manuscript are proof-of-concept experiments to validate the use of ExBox to access developmental windows and provide spatial specificity of expression (see Figures 7 and 8), experiments that as Reviewer 1 states in point 2 “*increase the impact of the paper*”.

Reviewer 1

We thank this Reviewer for their positive comments on our study, including: “*… it is worth noting that this paper reads easily. The manuscript is well written, and figures are good in quality. The rationale and experimental design are well explained and easy to follow.*”, and that “*the main conclusion of the paper is that ExBoX efficiently works on an exclusion logic, and this is indeed largely supported by data.*” We have addressed this reviewer’s comments, suggestions and minor points below.

1) The characterization of the system relies on the quantification of GFP and/or Tomato expressing cells in various experimental design. It is worth noting that the authors chose to co-transfect (or co-electroporate) ExBoX vectors with vectors expressing CRE, FlpO, and an additional fluorescent protein under a constitutive promoter. Thus, the quantification relies on the relative GFP (ExBoX) / RFP (constitutive) expression, assuming 100% co-transfection and expression of all 4 plasmids. Staining of CRE and/or Flp by immunofluorescence would convincingly demonstrate co-expression of recombinases in all cells and strengthen the data.

© 2021. Published by The Company of Biologists under the terms of the Creative Commons Attribution License (https://creativecommons.org/licenses/by/4.0/).

We agree with the reviewer in that this would be a nice addition to the data. It is worth noting that, for Figures 1 through 4, and new Figure 7, all the constructs expressing the OFF recombinases are bicistronic and express a fluorescent reporter from the same promoter. This was not very clear in the initial manuscript, except for the legend for Figure 4. We have now described this better in the main text and the other figure legends. This should address part of the concern raised by the reviewer.

In addition, after optimizing immunofluorescence against FlpO, we have repeated the in vitro experiments and stained for Cre and FlpO in different experimental settings to detect the On recombinase. The new results are unsurprisingly very similar to what we observed by direct fluorescence. Unfortunately, we were limited by the reagents currently available to visualize these recombinases in vivo. We have had limited success with Cre antibodies, and for FlpO we haven't had any success at all.

It is nevertheless important to note that if the co-transfection were inefficient, we would be underestimating the number of cells expressing the constructs in the On condition. When we co-transfect On recombinase + loading fluorescent reporter with the CreON- FlpOFF or FlpON-CreOFF constructs we detect co-expression in about 90 to 100% of the cells expressing the loading reporter (Figures 1-4). This suggests 2 things:

a) That the activation of the ExBox constructs is extremely high.
b) The co-transfection efficiency is high, otherwise the proportion of cells expressing both reporters would be much lower.

2) Another important aspect of ExBoX characterization is the temporal resolution of the system. Fate-mapping experiments using an exclusion system are a powerful tool to access temporal windows associated to specific developmental events, for example. This is largely discussed by the authors but one can regret that these experiments are not included in this manuscript. The model presented in Figure 8 could have easily been demonstrated using the FlpON-CreOff-TdTomato ExBoX vector expressed in any CRE- ERT2 mouse or cell line, together with constitutive FlpO expression. Thus, the authors would be able to easily demonstrate and quantify signal extinction after CRE induction by tamoxifen. Actually knowing if Tomato is turned off in a matter of hours, or in a matter of days, would make an important difference for the feasibility of temporally-controlled fate-mapping experiments. Thus, this additional experiment is technically feasible and would increase the impact of the paper.

This is a great suggestion. We now demonstrate that in a cell culture setting we can turn Off expression 4 days after tamoxifen treatment (Figure 7C). We go on to show that we can achieve a similar result in vivo 10 days after treatment, but we have not tried much shorter time points (Figure 7E, F). Finally, we now provide a proof-of-concept experiment for both spatial and temporal control of expression by ExBoX by performing sequential injections of AAVs driving the On and Off recombinase in the cortex (Figure 8). We visualize expression of the ExBoX constructs 3 weeks after injection of the Off recombinase, since expression driven by AAVs normally peaks between 3-4 weeks after injection. We would expect to be able to turn off expression in much shorter time- frames by using genetically encoded recombinase lines, but we did not have those at hand. Overall, these results provide strong validation for the ability of ExBoX to regulate expression in time and space. To our knowledge, this type of experiment demonstrating tight temporal and spatial control of expression has not been done for any of the other AAV-driven Boolean exclusion tools available (Fenno et al., 2014; Fenno et al., 2020).

From our initial co-expression experiments, we know that expression can be completely shut off in vivo in two days (Figure 4). It is important to note that even with a highly efficient system, it is unlikely that protein expression will be turned off within a few hours after recombination, but this will be ultimately dependent on mRNA and protein stability for each gene of interest. Thus, we think our system will be most powerful when studying developmental events that take place over days or weeks. We now discuss this in the Discussion section.

3) Finally, experiments in Figures 5-7 are not quantified. It appears that there are a few escapers from the exclusion system in vivo: see for example Figure 5F, figure 6F and Figure 7S-T. The authors should discuss this, and quantify the % of cells remaining ON. Do these escapers cells result from incomplete co-infection with CRE and FlpO AAVs? For example in Figure 5F even a 95%

© 2021. Published by The Company of Biologists under the terms of the Creative Commons Attribution License (https://creativecommons.org/licenses/by/4.0/).

co-infection rate would result in a few Tomato+ neurons. This can be detected by CRE immunostaining to check if the Tomato expressing neurons are CRE negative. Alternatively, this could stem from late recombination by the OFF recombinase, and slow elimination of the fluorescent protein.

We agree with the reviewer in that we can provide better quantification for those experiments. We now present the % of escapers for each of the ON+OFF conditions. As mentioned above, we tried co-staining for the recombinases, but we have had a hard time detecting them in vivo. We now discuss the different possibilities for why we observe more escapers in the AAV experiments than in the IUE and in vitro experiments extensively.

<u>Minor comments</u>
We found all these comments very valuable and were addressed by editing the text and/or figures.

-The authors used a synapsin promoter whith is a neuron-specific, weak and late promoter. If I understand correctly immuneamplification of the signal with anti-GFP and anti-DsRed has been applied to in vivo experiments. The authors could clarify this point and maybe provide one image without amplification so future users of ExBoX can evaluate the feasibility of in vivo experiments without amplification (ie. live imaging, etc...)
It has been made clearer in the figure legends which images have been amplified and which have not. Since AAVs were used the signal is robust and very little difference is seen between amplified and unamplified signals. We have included unamplified images in Figure S5.

-Table 1: the left column could be more clear. There's a confusion between recombinase expression, as indicated in the first line (column header) and the vector name/experimental system, referenced in the first column. Using the same nomenclature as in the manuscript (CreON-FlpOff-EGFP ExBoX, and FlpON-CreOff- TdTomato ExBoX) would be clearer.

The table has been modified to make it clearer and more consistent with the rest of the paper.

-Figure 1 and 2: vector design representation could be improved. I would strongly suggest to use distinct symbols for Lox and Frt sites. Also being consistent in color scheme is important: Figure 1A, black arrowheads are Frt sites, but Figure 2A, black arrowheads are Lox2722 sites. Figure 1A, LoxP and Lox2722 sites are represented as red and blue arrowheads, yet CRE expression/recombination is indicated as a black arrow. Frt sites are represented as black arrowheads, yet Flp Eexpression/recombination is indicated ad a red arrow.
Figure 1A, CRE is black arrow and FlpE is red arrow, yet Figure 2A, CRE is red arrow and FlpE is black arrow.
Recombinase site colors have now been made consistent across figures. We preferred not to change the shapes between the lox and frt sites since triangles are useful to depict orientation of the sites within the construct. For that reason, triangles are a common convention and we feel that switching for other shapes may cause more confusion.

-Figure lettering seems to be mixed in the first paragraph of 'Validation of ExBoX AAVs in vivo' (figure 5).
Figure lettering was indeed mixed and has been corrected.

<u>Significance:</u>
The purpose of ExBoX is to provide a simple and efficient exclusion logic (AND NOT) reporter system. The authors make it clear that this strategy has been developed previously and thus ExBoX is not a novel method but rather a refinement, providing a simpler construct design that might be easier to use. While this reviewer does not have any hands-on experience with AAV vectors use and virus production, the author's argument seem valid and thus my impression would be that ExBoX provides some technical advance and would be useful for the scientific community. Of note the authors validate ExBoX in neurons, but the same system could easily be adapted to other tissues simply by changing the promoter of the ExBoX construct. Thus, the system might be of interest outside of the neuroscience community.

We would like to thank the reviewer for stating that ExBox could provide some "*technical advance and would be useful for the scientific community*", and that "*could easily be adapted to other*

© 2021. Published by The Company of Biologists under the terms of the Creative Commons Attribution License (https://creativecommons.org/licenses/by/4.0/).

tissues simply by changing the promoter of the ExBoX construct. Thus, the system might be of interest outside of the neuroscience community."

We are in agreement with the reviewer in that similar strategies were designed before, and as stated by them, we make this clear through-out the paper. That said, such viral tools were not validated in vivo to the extent that we validate ours in this revised manuscript. The goal of our new ExBox strategy was to make the use of these tools universal, by providing a simpler design and a more efficient use of vector space. In addition to redesigning a Boolean exclusion system we generated new viral CreOn- FlpOff constructs for modulation of neuronal activity using Kir2.1, NachBac and TeNT. All these constructs are being deposited in Addgene and will be available to the scientific community. Additional considerations about impact and novelty are discussed in response to Reviewer 2.

Reviewer 2
We thank reviewer 2 for their comments. We were glad to see that this reviewer didn't have any technical or methodological concerns. We also thank the reviewer for bringing those studies to our attention. We will now cite them in the main text of the manuscript.

The only issue raised by Reviewer 2 was about novelty. We tried to make it clear through-out our manuscript that we are building on previous work (Fenno et al., 2014 among other citations) to design a simpler, more efficient and universal tool to drive tightly controlled expression using Boolean exclusion logic. By using viruses with a very simple modular design we believe we achieved this goal. Anyone with any interest in using these tools can clone their gene of interest in these vectors in a simple step. In addition, promoters can be easily replaced to drive expression in a multiplicity of tissues and model organisms for a very low cost. This also adds to the universality of the tools. Furthermore, as mentioned in the discussion, the modularity of the system would make it easy to adapt to other recombinases (e.g. DRE), or to add additional sites to achieve an AND NOT NOT logic. Finally, we also introduce new viral CreOn-FlpOff constructs for Kir2.1, NachBac and TeNT that will have broad applicability. Taken together we believe that the importance of such work in driving science forward resides not only in adding "new arrows to the quiver", but in making them easier to use, easier to build upon, easier to disseminate, and easier to adapt to different animal models and organs.

That said, the design of the ExBox AAVs is actually novel. To our knowledge, there are no published or publicly available AAVs using this strategy. We truly believe that the papers cited by Reviewer 2 don't take anything away from the impact of our work. The two papers describing the generation of TRAP tools, while using a very similar design to our CreOn-FlpOff construct, involve the generation of *knock in* mice, and the overall goal of the approach is exactly the opposite of that of ExBoX: control the silencing of endogenous genes. In addition, the design of the FlpOn-CreOff constructs is completely new. We now cite these two papers in the introduction as examples of combinatorial approaches. The third paper cited by Reviewer 2, which was published while this manuscript was in preparation, uses an elegant but different design (a hybrid *lox* system) to achieve something very similar to what we propose. However, as stated by the reviewer, it is expected that ExBoX system would be more efficient (in addition to being more modular, more widely applicable and adaptable). This paper is now mentioned in the discussion section.

Reviewer 3
We are very pleased with the extremely positive comments this reviewer has made on the impact, significance, and quality of our study. We agree with Reviewer 3 in that ExBox is "*ideal for viral packaging which is vital for use in vivo*." It is our intention to disseminate these tools for immediate use by the scientific community. We have already started the process of depositing them at Addgene.

© 2021. Published by The Company of Biologists under the terms of the Creative Commons Attribution License (https://creativecommons.org/licenses/by/4.0/).

Second decision letter

MS ID#: JOCES/2020/257212

MS TITLE: ExBoX: a simple Boolean exclusion strategy to drive expression in neurons

AUTHORS: Teresa Ubina, Tyler Vahedi-Hunter, Will Agnew-Svoboda, Akshay Gupta, Wenny Wong, Vijayalakshmi Santhakumar, and Martin Miguel Riccomagno
ARTICLE TYPE: Tools and Resources

Thank you for sending your manuscript to Journal of Cell Science through Review Commons.

I am happy to tell you that your manuscript has been accepted for publication in Journal of Cell Science, pending standard ethics checks.

© 2021. Published by The Company of Biologists under the terms of the Creative Commons Attribution License (https://creativecommons.org/licenses/by/4.0/).
